# From Cyanobacteria to Human, MAPEG-Type Glutathione-S-Transferases Operate in Cell Tolerance to Heat, Cold, and Lipid Peroxidation

**DOI:** 10.3389/fmicb.2019.02248

**Published:** 2019-09-27

**Authors:** Xavier Kammerscheit, Franck Chauvat, Corinne Cassier-Chauvat

**Affiliations:** Institute for Integrative Biology of the Cell (I2BC), CEA, CNRS, University of Paris-Sud, Université Paris-Saclay, Gif-sur-Yvette, France

**Keywords:** *Synechocystis* PCC 6803, microsomal glutathione transferase, mGST2, mGST3, temperature stresses, heterologous complementation, knockout mutant, membrane stress

## Abstract

The MAPEG2 sub-family of glutathione-S-transferase proteins (GST) has been poorly investigated *in vivo*, even in prokaryotes such as cyanobacteria the organisms that are regarded as having developed glutathione-dependent enzymes to protect themselves against the reactive oxygen species (ROS) often produced by their powerful photosynthesis. We report the first *in vivo* analysis of a cyanobacterial MAPEG2-like protein (Sll1147) in the model cyanobacterium *Synechocystis* PCC 6803. While Sll1147 is dispensable to cell growth in standard photo-autotrophic conditions, it plays an important role in the resistance to heat and cold, and to n-*tert*butyl hydroperoxide (n-*t*BOOH) that induces lipid peroxidation. These findings suggest that Sll1147 could be involved in membrane fluidity, which is critical for photosynthesis. Attesting its sensitivity to these stresses, the Δ*sll1147* mutant lacking Sll1147 challenged by heat, cold, or n-*t*BOOH undergoes transient accumulation of peroxidized lipids and then of reduced and oxidized glutathione. These results are welcome because little is known concerning the signaling and/or protection mechanisms used by cyanobacteria to cope with heat and cold, two inevitable environmental stresses that limit their growth, and thus their production of biomass for our food chain and of biotechnologically interesting chemicals. Also interestingly, the decreased resistance to heat, cold and n-*t*BOOH of the Δ*sll1147* mutant could be rescued back to normal (wild-type) levels upon the expression of synthetic MAPEG2-encoding human genes adapted to the cyanobacterial codon usage. These synthetic hmGST2 and hmGST3 genes were also able to increase the *Escherichia coli* tolerance to heat and n-*t*BOOH. Collectively, these finding indicate that the activity of the MAPEG2 proteins have been conserved, at least in part, during evolution from (cyano)bacteria to human.

## Introduction

Glutathione, the highly abundant (1–10 mM) tripeptide L-glutamyl-L-cysteinyl-L-glycine (Lu, 2013), plays an important role in the protection against oxidative and metabolic stresses in most organisms. The reduced (major) form (GSH) of glutathione maintains reduced state of the intracellular compartment(s) and donates electrons to various enzymes (Fahey, 2013; Lu, 2013), such as the glutathione-S-transferases (GST) that operate in the detoxification of reactive oxygen species (ROS), xenobiotics, and/or heavy metals (Yadav, 2010; Noctor et al., 2012). The resulting oxidized form of glutathione, the dimeric disulfide form (GSSG), is then reduced back to GSH by various factors, such as the NADPH-using enzyme glutathione reductase (GR) that occurs in many (Couto et al., 2016), but not all organisms such as the presently studied model cyanobacterium *Synechocystis* sp. PCC 6803 (Marteyn et al., 2009; Naraimsamy et al., 2013). The superfamily of GSTs enzymes (EC 2.5.1.18) can be divided in three categories: the cytosolic GSTs, the mitochondrial GSTs and the microsomal GSTs also designated as MAPEGs (membrane-associated proteins involved in ecosanoïd and glutathione metabolism) (Oakley, 2011).

Mammalian membrane-associated proteins involved in ecosanoïd and glutathione metabolisms have different functions and low sequence similarities. They define six types of proteins namely: (i) mGST1, (ii) mGST2 and (iii) mGST3, (iv) the 5-lipoxygenase-activating protein (FLAP), (v) the prostaglandin E synthase (PGES) and (vi) the leukotriene C_4_ (LTC_4_) synthase, which can be grouped in two families: MAPEG1 (mGST1/PGES) and MAPEG2 (mGST2/mGST3/FLAP/LTC_4_ synthase) (for reviews see Jakobsson et al., 2000; Bresell et al., 2005; Pearson, 2005; Deponte, 2013). MAPEG homologs identified in insects (*Drosophila melanogaster*, and *Anopheles gambiae*) can be placed in the MAPEG1 family (Bresell et al., 2005), while MAPEG homologs found in fungi (*Aspergillus nidulans*) and plants (*Arabidopsis thaliana*, *Oryza sativa*, and *Ricinus communis*) belong to MAPEG2 (Bresell et al., 2005). MAPEG1 and MAPEG2 members were found in prokaryotes but not in archae (Bresell et al., 2005; Pearson, 2005).

Membrane-associated proteins involved in ecosanoïd and glutathione metabolism proteins are not merely “membrane associated” proteins as proposed by their acronym, but in fact integral membrane proteins (Deponte, 2013). They contain 4 transmembranes helices as was suggested by their hydropathy plots (Jakobsson et al., 1999; Bresell et al., 2005) and verified in the X-ray crystallography of the mammalian proteins: mGST1 (Holm et al., 2002, 2006), LTC_4_ synthase (Ago et al., 2007) and PGES (Sjögren et al., 2013). *Furthermore, the trimeric state was revealed by* X-ray crystallography for mGST1 (Hebert et al., 1995; Ålander et al., 2009). Each monomer were found to bind on GSH molecule but only one subunit is catalytically active in both rat mGST1 and human mGST2 (Ålander et al., 2009; Ahmad et al., 2015).

Mammalian MAPEGs use a GSH-dependent isomerase activity to operate in the biosynthesis of leukotrienes (FLAP and LTC_4_ synthase) or prostaglandin E (PGES) (Morgenstern et al., 2011), (GPI) (Deponte, 2013) that are required for allergic (Ago et al., 2007) and inflammatory (Sjögren et al., 2013) responses. MAPEGs also play a role in detoxification (Johansson et al., 2010), using a GSH-transferase activity (Deponte, 2013) as well as the GSH-dependent reduction of organic fatty acid and lipid hydroperoxides through their peroxidase activity (Mosialou et al., 1995; Johansson et al., 2010; Deponte, 2013; Zhang et al., 2017).

Unlike the human mGST1 (hmGST1), which operates in lipid peroxidation (Johansson et al., 2010) and tumorigenesis and drug resistance (for review see Morgenstern et al., 2011), the precise *in vivo* role of (human) hmGST2 and hmGST3 is not well described though they could be interesting targets for drug development. Very interestingly, hmGST2 and hmGST3 share amino-acids sequence homology with MAPEG-like proteins from simple and fast growing prokaryotes, such as *Escherichia coli* and the model cyanobacterium *Synechocystis* PCC 6803. These *E. coli* and *Synechocystis* PCC 6803 MAPEG-like genes were cloned in an *E. coli* expression vector, and the MAPEG-like proteins were purified (partially in the case of the *Synechocystis* PCC 6803 protein) and appeared to catalyze the conjugation of 1-chloro-2,4-dinitrobenzene (CDNB) with GSH, thereby showing that GST activity can be regarded as a common denominator for a majority of MAPEG members throughout the kingdoms of life (Bresell et al., 2005). These findings are interesting because cyanobacteria are attractive organisms to study the role of GSTs and their selectivity/redundancy. They are regarded as the inventor of the oxygenic photosynthesis (Archibald, 2009; William Schopf, 2011) and of the GSH synthesis and dependent enzymes to protect themselves against the ROS they (often) produce when their active photosynthesis is altered by stresses (light, temperature, and metals) (William Schopf, 2011).

In the frame of our long-term interest in stress responses in cyanobacteria (for a review see Cassier-Chauvat and Chauvat, 2014) we carried out the first (to our knowledge) *in vivo* analysis, of the MAPEG protein of *Synechocystis* sp. PCC 6803 (hereafter *Synechocystis*). We report that the *Synechocystis* MAPEG protein (designated Sll1147 in CyanoBase) plays a prominent role in the cell tolerance to heat- and cold-temperatures and the protection against lipid peroxidation. The Δ*sll1147* deletion mutant lacking Sll1147 undergoes a transient sequential accumulation of peroxidized lipids and then of GSH and GSSG. We also demonstrate that the stress-tolerance of the Δ*sll1147* mutant can be restored following the production of hmGST2 and hmGST3 the human MAPEG counterparts of Sll147. This finding shows that (at least some) MAPEG functions have been conserved throughout evolution and can be (easily) studied in *Synechocystis*.

## Results

### The Sll1147 MAPEG-Like GST Is Dispensable for the Photoautotrophic Growth of *Synechocystis*

**FIGURE 1:**
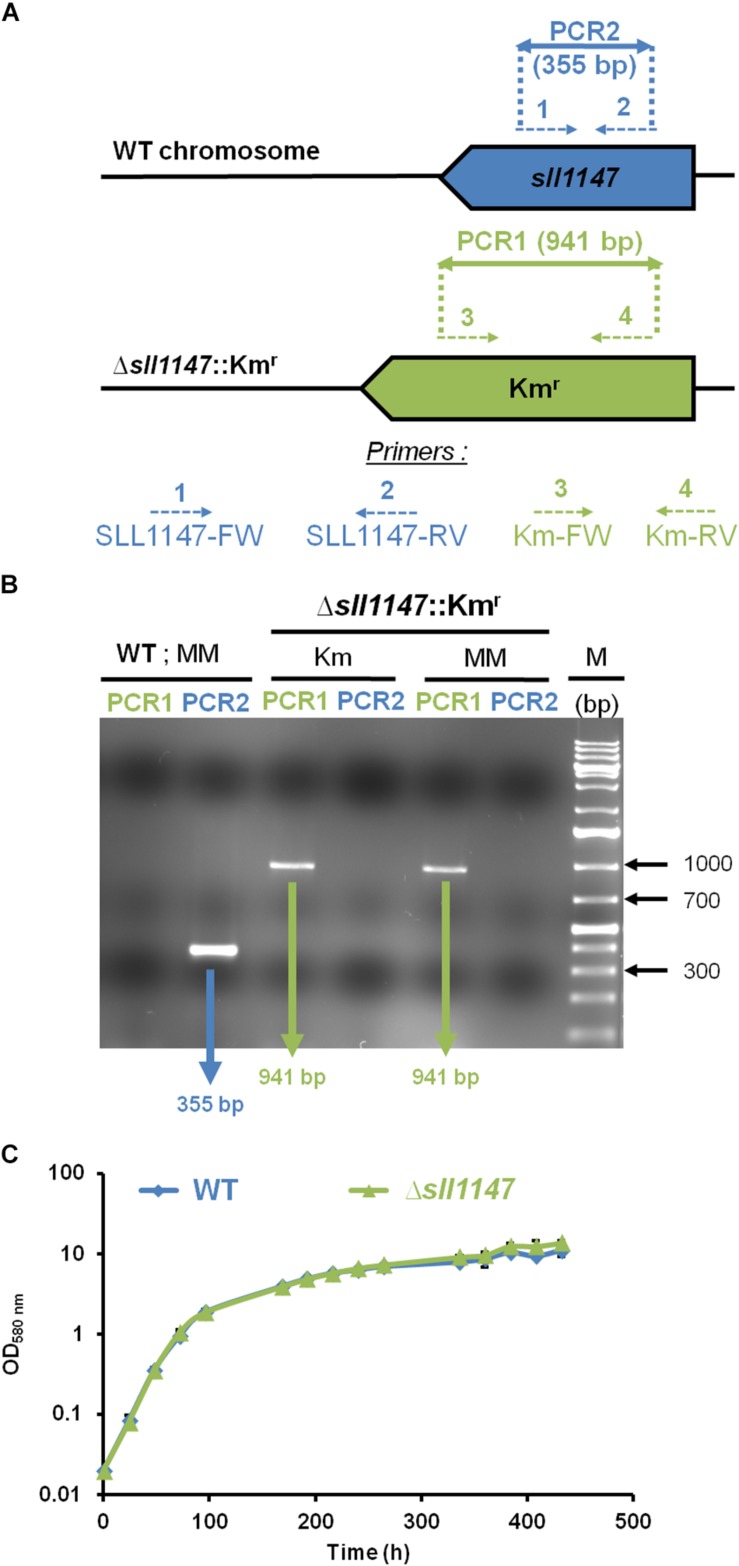
The *sll1147* gene is dispensable for the photoautotrophic growth of *Synechocystis*. **(A)** PCR analysis of the *sll1147* chromosome locus in *Synechocystis* WT strain and the Δ*sll1147*:Km^*r*^ mutant constructed in this study. The genes are represented by colored arrows (*sll1147*^+^, blue; Km^*r*^, green) pointing into the direction of their transcription. The same color code is used to represent the PCR oligonucleotides primers (dotted arrows, see Supplementary Table S1 for their sequence) and the corresponding PCR products (double arrows) typical of the presence of WT (*sll1147*^+^ and Km^*S*^) or mutant (Δ*sll1147*: Km^*r*^) chromosome copies. **(B)** Typical UV-light image of the agarose gel showing the PCR products corresponding to the genes *sll1147*^+^ (PCR2, blue) or Km^*r*^ (PCR1, green) generated from WT and Δ*sll1147*: Km^*r*^ cells grown in absence (MM) or presence (Km) of the selective antibiotic. M (marker DNA) stands for the GeneRuler 1 kb Plus DNA Ladder (Thermo Fisher Scientific). Note that WT cells harbor only WT chromosomes while Δ*sll1147*:Km^*r*^ mutant cells possess only Δ*sll1147*:Km^*r*^ chromosomes. **(C)** Typical growth curves of the WT strain and *sll1147* mutant growing under standard photoautotrophic conditions.

To analyze *in vivo* the role of the Sll1147 GST, we constructed the Δ*sll1147*:Km^*r*^ deletion mutant, using the standard gene replacement procedure that comprises three main steps. First, the DNA region containing *sll1147* surrounded by its 300 bp flanking DNA regions was amplified by PCR using specific oligonucleotides primers (Supplementary Table S1). Second, the *sll1147* protein-coding sequence was replaced by a transcription-terminator-less kanamycin resistance (Km^*r*^) gene for selection, while preserving the 300 bp of *sll1147* flanking DNA regions for homologous recombination that mediate targeted gene replacement upon transformation to *Synechocystis* (Labarre et al., 1989). Third, the resulting deletion cassette (Supplementary Table S2) was introduced in *Synechocystis* by transformation (Labarre et al., 1989). A few Km^*r*^ transformants were selected and analyzed by PCR with specific oligonucleotide primers (Figures 1A,B and Supplementary Table S1) to verify that the Km^*r*^ marker gene had properly replaced *sll1147* in the *Synechocystis* chromosome, thanks to homologous DNA recombination occurring in the 300-bp *sll1147*-flanking DNA region shared by the Δ*sll1147*:Km^*r*^ incoming DNA and the *Synechocystis* recipient chromosome which is polyploid [it occurs at about ten copies per cell (Labarre et al., 1989)].

All Δ*sll1147*:Km^*r*^ transformants growing in standard photoautotrophic conditions in the presence of kanamycin possessed only Δ*sll1147*:Km^*r*^ chromosomes (Figure 1B, see the presence of the 941 bp PCR product characteristic of Δ*sll1147*:Km^*r*^ chromosomes) but no WT chromosomes [Figure 1B, see the absence of a 355 bp PCR band typical of WT (*sll1147*^+^) chromosomes]. The absence of WT chromosome copies (*sll1147*^+^, i.e., Km^*S*^) in the Δ*sll1147*:Km^*r*^ mutant was confirmed by growing it for multiple generations in absence of Km to stop counter-selecting possible WT (*sll1147*^+^ and Km^*S*^) chromosome copies, which could have escape detection, prior to the PCR assays. Again the Δ*sll1147*:Km^*r*^ mutant possessed only Δ*sll1147*:Km^*r*^ chromosomes (Figure 1B, see the presence of the 941 bp PCR product and the absence of a 355 bp PCR band). Together, these data demonstrate that *sll1147* is non-essential for the standard photoautotrophic growth of *Synechocystis*.

### The Sll1147 MAPEG-Like GST Operates in the Tolerance of *Synechocystis* to n-*tert*butyl Hydroperoxide

**FIGURE 2:**
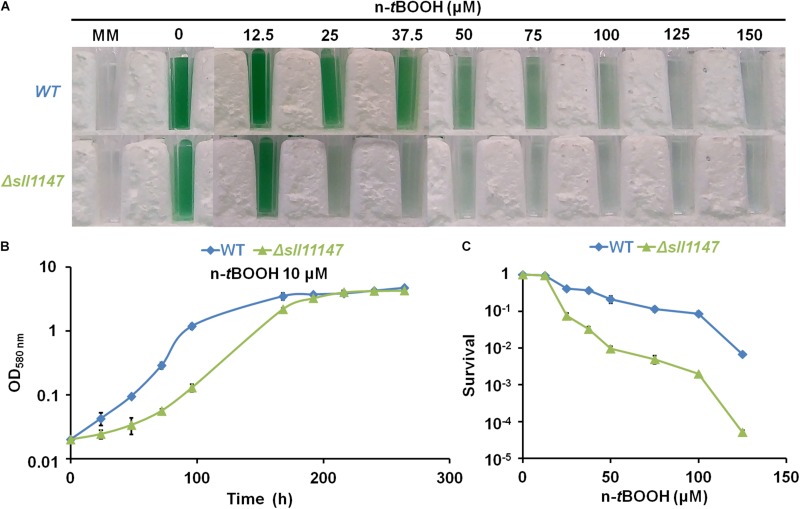
The Δ*sll1147* mutant is sensitive to n-*tert*butyl hydroperoxide. **(A)** Ten milliliters of mid-log-phase cultures (initial OD_580__nm_ = 0.1) were incubated for 72 h in liquid mineral medium (MM) under standard photoautotrophic conditions with or without the indicated concentrations of n-*t*BOOH, prior to transfer in spectrophotometric cuvette and photography. **(B)** Typical growth curve of the WT strain and Δ*sll1147* mutant incubated in liquid MM containing 10 μM n-*t*BOOH. **(C)** Typical survival of WT and Δ*sll1147* cells exposed for 72 h to various n-*t*BOOH concentrations. Data shown in **(B,C)** are expressed as the mean ± standard deviation (SD) of three experiments.

Recently, we showed that the Sll1545 and Slr0236 GSTs, distantly related to the presently studied MAPEG-like GST Sll1147, operate (negatively) in the tolerance to hydrogen peroxide (Kammerscheit et al., 2019) a ROS frequently generated by the cell metabolism (Imlay, 2013). Consequently, having a strong interest in the selectivity/redundancy of the role of GSH-dependent enzymes (Picciocchi et al., 2007; Michaut et al., 2008; Marteyn et al., 2009, 2013), we have tested the influence of hydrogen peroxide (H_2_O_2_) and n-*tert*butyl hydroperoxide (n-*t*BOOH), which induces lipid peroxidation (Andrade et al., 2018), on the fitness of the *Synechocystis* WT strain and the Δ*sll1147*:Km^*r*^ mutant. Both the growth and survival of the Δ*sll1147*:Km^*r*^ mutant (hereafter Δ*sll1147*) were decreased by n-*t*BOOH (Figures 2B,C) and H_2_O_2_ (Supplementary Figure S1), as compared to the WT strain. Collectively, these data suggest that Sll1147 operates in the protection against the two peroxides H_2_O_2_ and n-*t*BOOH.

### In Response to n-*tert*butyl Hydroperoxide (*n*-*t*BOOH), the Δ*sll1147* Sensitive Mutant Transiently Accumulates Peroxidized Lipids and Then Glutathione

To analyze the influence of n-*t*BOOH on the Δ*sll1147* sensitive mutant and the WT control strain, we measured the malondialdehyde (MDA) content as a marker of lipid peroxidation, using the standard thiobarbituric acid reactive substances (TBARS) assay (Zeb and Ullah, 2016). As GSTs are generally involved in glutathione-dependent reactions, we also measured the levels of the reduced, oxidized and total glutathione (GSH, GSSG, and GS_*total*_ = GSH + GSSG) using the relevant standard assay (Akerboom and Sies, 1981) as we previously described (Kammerscheit et al., 2019).

**FIGURE 3:**
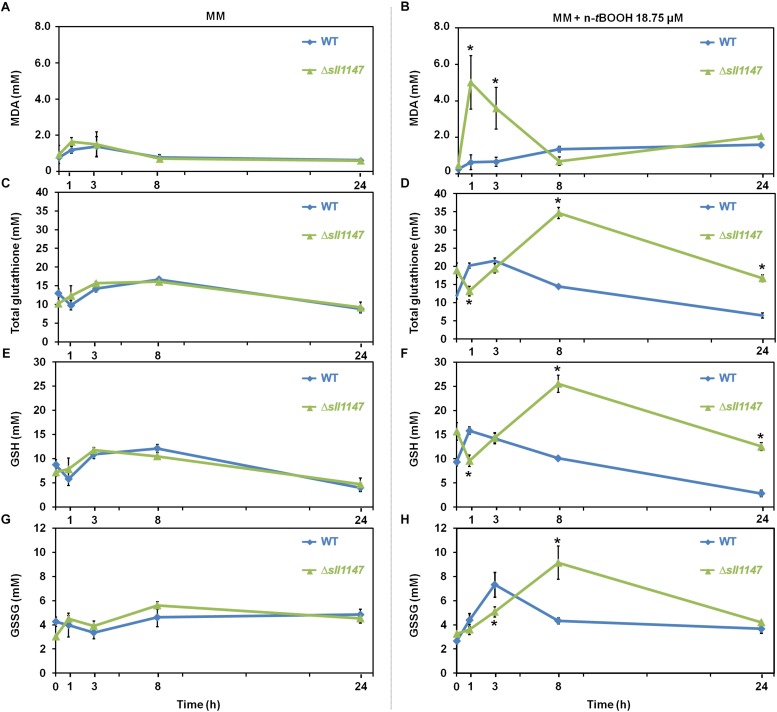
The Δ*sll1147* sensitive mutant challenged by n-*t*BOOH, transiently accumulates peroxidized lipids and then glutathione. Kinetic analysis of the influence of n-*tert*butyl hydroperoxide (n-*t*BOOH) on the abundance of peroxidized lipids, measured as the MDA content **(A,B)**, total glutathione **(C,D)**, GSH **(E,F)**, and GSSG **(G,H)** in *Synechocystis* WT and Δ*sll1147* cells. The data are expressed as the mean ± SD of three assays and ^∗^ indicates significant difference between mutant and WT (*t*-test, *P* < 0.05).

In the WT strain, the level of MDA was almost unaffected by (18.75 μM) n-*t*BOOH (Figure 3B) while GSH and GSSG were weakly (about twofold) and transiently increased at 1 h (GSH) and 3 h (GSSG), before returning to their normal levels at 24 h (Figures 3F,H). These data are consistent with the WT strain being little affected by n-*t*BOOH [it was resistant up to 25 μM; (Figure 2)]. By contrast, the Δ*sll1147* mutant challenged by n-*t*BOOH highly and rapidly accumulated MDA (within 1 h), the level of which decreased down to the unstressed level (at 8 h, Figure 3B). Concomitantly to the MDA decline (between 1 and 3 h), the mutant Δ*sll1147* strongly accumulated both GSH and GSSG (up to 25 and 10 mM, respectively), that subsequently returned to the unstressed level (at 24 h, Figures 3B,F,H). The strong and sequential accumulation of the stress indicator MDA (at 1 h) and then (at 8 h) of GSH (detoxification) and GSSG (generated upon the GSH-mediated detoxification) are consistent with the high sensitivity of the Δ*sll1147* mutant to n-*t*BOOH (Figure 2). Altogether, these findings indicate that Sll1147 normally operates in the protection against peroxidized lipids elicited by n-*t*BOOH, using a process that transiently accelerates the synthesis of reduced glutathione (GSH) which is subsequently oxidized in glutathione disulfide (GSSG). In contrast, Δ*sll1147* cells challenged by H_2_O_2_ did not significantly modify their level of GSH and GSSG as compared to WT cells (Supplementary Figure S2), indicating that the Sll1147 and glutathione-dependent response is somehow more “specific” to peroxidized lipids than to other kind of ROS, as observed with mGST1 substrates (Mosialou et al., 1995).

### The Sll1147 MAPEG-Like GST Operates in the Tolerance of *Synechocystis* to Both Heat and Cold Stresses

**FIGURE 4:**
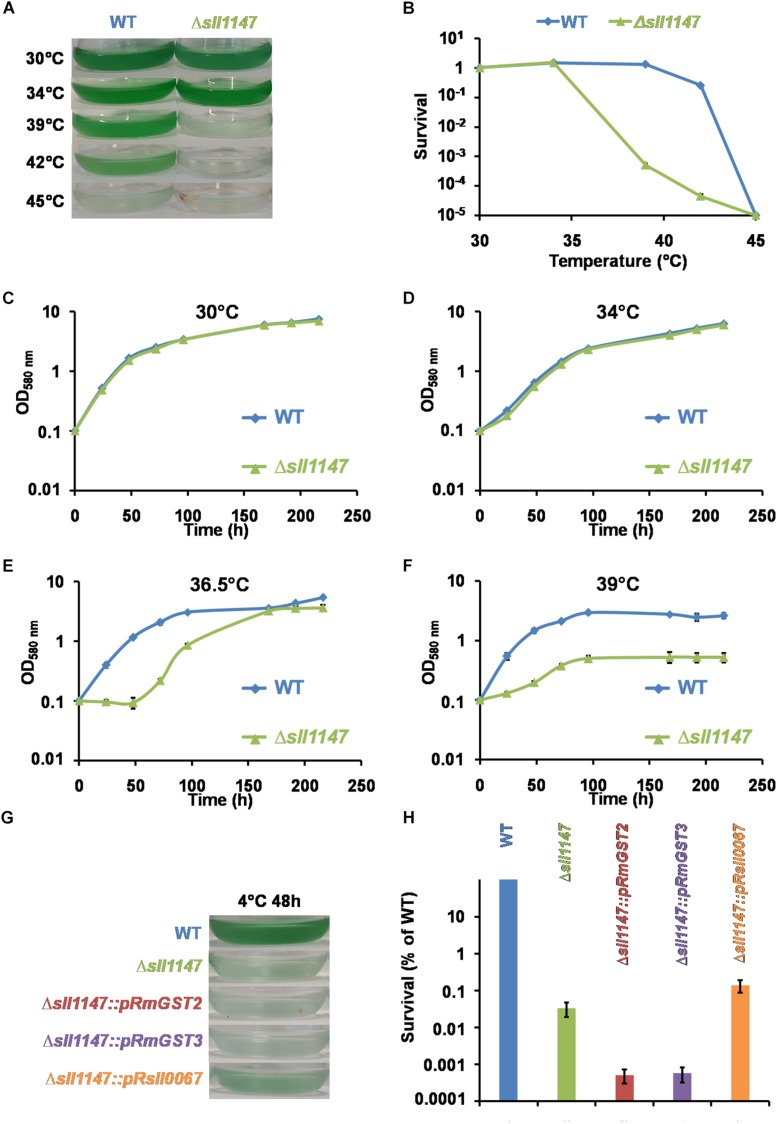
The Sll1147 MAPEG-like GST is involved in the *Synechocystis* tolerance to high and low temperatures. Ten milliliters of mid-log-phase cultures (initial OD_580__nm_ = 0.1) were incubated in liquid MM for 24 h under standard light (2500 lux; 31.25 μE.m^– 2^.s^–1^) at the indicated temperatures, prior to photographing the culture flasks **(A)** and survival analysis **(B)**. **(C–F)** Growth curves of WT and Δ*sll1147* strains incubated in liquid MM under standard light (2500 lux; 31.25 μE.m^–2^.s^–1^) at various temperatures: 30°C **(C)**, 34°C **(D)**, 36.5°C **(E)**, and 39°C **(F)**. **(G)** Ten milliliters of mid-log-phase cultures (initial OD580nm = 0.1) were incubated in liquid MM for 48 h under standard light (2500 lux; 31.25 μE.m^–2^.s^–1^) at 4°C and then 24 h at 30°C for recovery, before photographing the culture flasks. **(H)** Survival of WT strain and Δ*sll1147*, Δ*sll1147:pRmGST2*, Δ*sll1147:pRmGST3*, and Δ*sll1147:pRsll0067* mutants exposed for 48 h to 4°C. Data shown in **(B–F,H)** are expressed as the mean ± SD of three experiments.

Several lines of evidence prompted us to test the influence of the presence (WT) and absence of Sll1147 (Δ*sll1147* mutant) on the tolerance of *Synechocystis* to high and low temperatures. Like other photosynthetic organisms, cyanobacteria are frequently exposed to fluctuations in temperatures triggered by the alternation of seasons, the day-night cycles, and the passages of clouds that filter sunlight. Furthermore, it is known that (i) temperature stress can trigger lipid peroxidation; (ii) mutant impaired in the defense against lipid peroxidation are temperature sensitive, and (iii) glutathione operates in the resistance to temperature stress and lipid peroxidation (Hasanuzzaman et al., 2017). Consequently, we have tested and compared the influence of high (39°C) and low (4°C) temperatures on the growth and/or survival of the WT and Δ*sll1147* strains of *Synechocystis*. The fitness of the two strains was similar at 30°C (the standard growth temperature of *Synechocystis*) and 34°C (Figures 4C,D). As compared to the WT strain, the growth and survival of the Δ*sll1147* mutant were decreased by high temperatures (Figures 4E,F). These data showed that Sll1147 is required for the tolerance to heat. We also found that the survival of the Δ*sll1147* mutant was strongly decreased after a 48 h exposure at 4°C (Figures 4G,H), showing that Sll1147 is also involved in the tolerance to low temperatures. To our knowledge this is the first evidence that a cyanobacterial GST (the MAPEG-like Sll1147 protein) operates in the protection against heat and cold stresses.

### The Heat-Sensitive Mutant Δ*sll1147* Exposed to a High Temperature Transiently Accumulates Peroxidized Lipids and Then Glutathione

**FIGURE 5:**
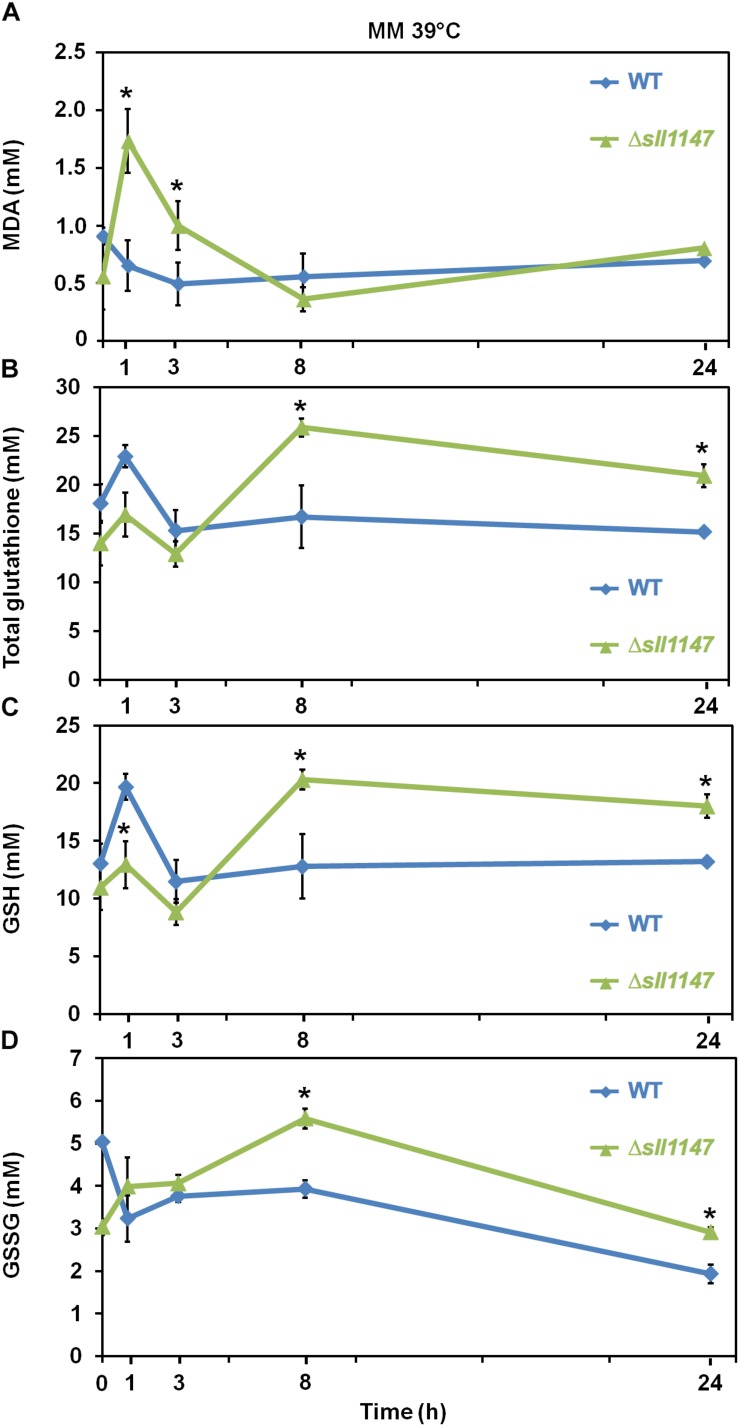
The Δ*sll1147* heat sensitive mutant challenged by heat transiently accumulates peroxidized lipids, GSH, and GSSG. Kinetic analysis of the influence of high temperature exposure (39°C) on the abundance of peroxidized lipids measured as MDA content **(A)**, total glutathione **(B)**, GSH **(C)**, and GSSG **(D)** in *Synechocystis* WT and mutant Δ*sll1147*. Data are expressed as the mean ± SD of three assays and ^∗^ indicates significant difference between mutant and WT (*t*-test, *P* < 0.05).

Heat is an important environmental stressor known to elicit lipid peroxidation in photosynthetic organisms where it leads to the formation of the secondary product, MDA that can be detected by TBARS assay [for example see, (Prasad et al., 2016) and references therein]. Thus, to analyze the influence of heat in *Synechocystis* WT strain and Δ*sll1147* mutant we measured in each strain the MDA content and the levels of the reduced and oxidized glutathione (GSH, GSSG and total glutathione). In accordance with its heat sensitivity the Δ*sll1147* mutant incubated at 39°C rapidly (within 1 h) accumulated MDA, which decreased down to the unstressed level (at 8 h, Figure 5A). Concomitantly to the MDA decline (particularly between 3 and 8 h), the Δ*sll1147* mutant accumulated both GSH and GSSG (up to about 20 and 5 mM, respectively), which remained high (GSH, Figure 5C) or decreased down to the unstressed level (at 24 h, GSSG, Figure 5D). Collectively, these data indicate that Sll1147 normally operates in the detoxification of peroxidized lipids elicited by heat, using a redox process that transforms reduced glutathione (GSH) in oxidized glutathione (GSSG).

### The Cold-Sensitive Mutant Δ*sll1147* Exposed to a Low Temperature Transiently Accumulates Peroxidized Lipids and Then Glutathione

**FIGURE 6:**
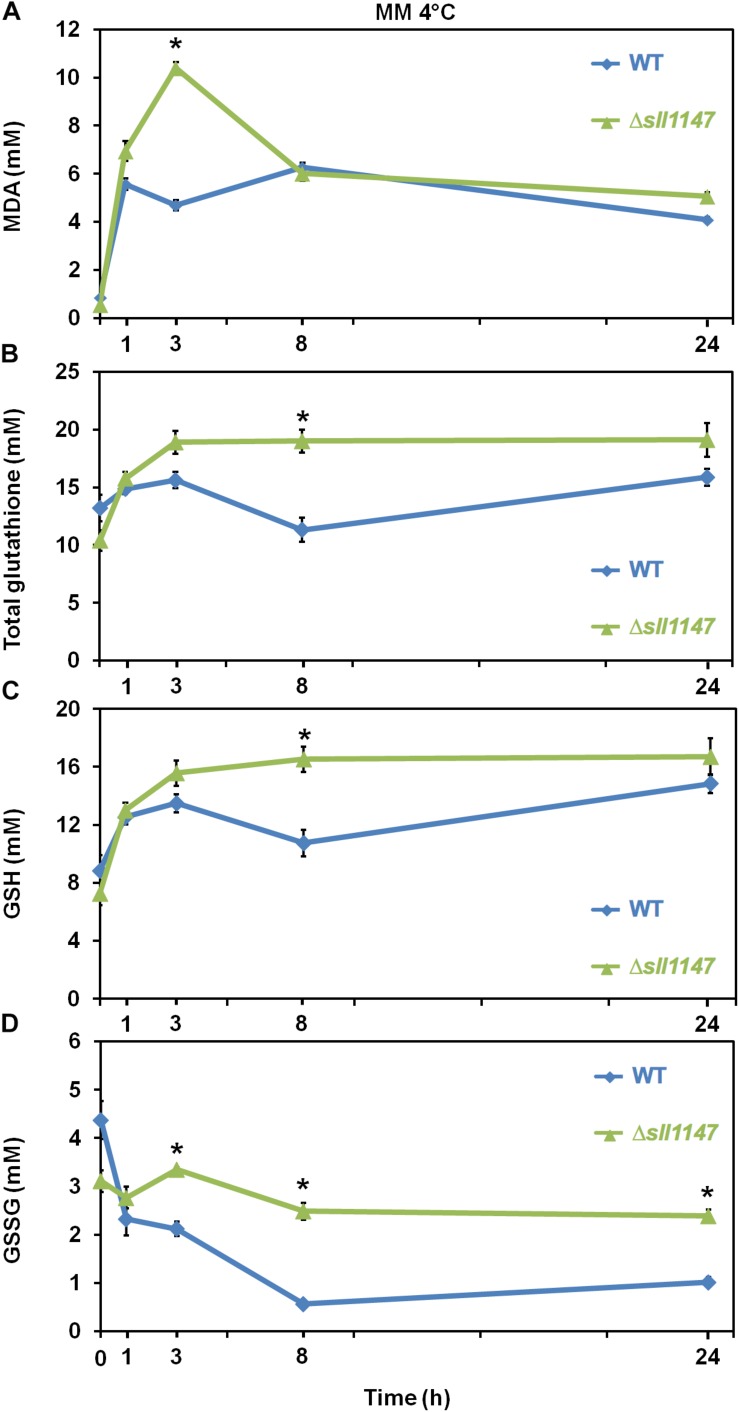
In response to cold, the Δ*sll1147* sensitive mutant transiently accumulates peroxidized lipids, GSH and GSSG. Kinetic analysis of the influence of cold temperature exposure (4°C) on the abundance of peroxidized lipids measured as MDA content **(A)**, total glutathione **(B)**, GSH **(C)**, and GSSG **(D)** in *Synechocystis* WT and mutant Δ*sll1147*. Data are expressed as the mean ± SD of three assays and ^∗^ indicates significant difference between mutant and WT (*t*-test, *P* < 0.05).

We also analyze the influence of cold in *Synechocystis* WT strain and Δ*sll1147* mutant on the level of reduced and oxidized glutathione (GSH and GSSG) and on the MDA content, because cold treatment modifies the content of total glutathione and increases ROS (H_2_O_2_ and O_2_^–^) and malondialdehyde levels (MDA) (Hasanuzzaman et al., 2017). In agreement with its cold sensitivity the Δ*sll1147* mutant incubated at 4°C rapidly (within 1 h) accumulated MDA which peaked at 3 h (up to 10 mM) and subsequently decreased down to the WT level (at 8 h, Figure 6A). The Δ*sll1147* mutant accumulated both GSH and GSSG (up to about 16 and 3 mM, respectively), which remained high (for both GSH and GSSG, Figures 6C,D) compared to the WT levels. Collectively, these data indicate that Sll1147 normally operates in the detoxification of peroxidized lipids elicited by heat, using a redox process that transforms reduced glutathione (GSH) in oxidized glutathione (GSSG).

### The Human hmGST2 and hmGST3 MAPEG Proteins, but Not the Unrelated Sll0067 *Synechocystis* GST Protein, Can Restore the Cell Tolerance to n-*t*BOOH and Heat of the *Synechocystis* Mutant Lacking the Sll1147 MAPEG-Like Protein

Previous bioinformatic studies showed that the cyanobacterial Sll1147 protein shares common features (amino-acid sequences and hydropathy plots) with the human mGST2 and mGST3 MAPEGs (hereafter hmGST2 and hmGST3) (Jakobsson et al., 1999). As the role of these hmGST2 and hmGST3 proteins in cell physiology is poorly understood, we tested whether they can operate in the tolerance to temperature and lipid peroxidation stresses, like their “(cyano)bacterial ancestor” Sll1147. Practically, we tested whether the hmGST2 and/or hmGST3 proteins can restore the tolerance to heat, cold and n-*tert*butyl hydroperoxide (n-*t*BOOH) in the *Synechocystis* Δ*sll1147* mutant lacking Sll1147. Therefore, we constructed the gene-complementation DNA cassettes that contain the following parts (Supplementary Table S2 and Supplementary Figure S3). A synthetic version of the *hmGST*2 or *hmGST3* genes adapted to the cyanobacterial codon usage and expressed from the efficient pR promoter (Mermet-Bouvier and Chauvat, 1994). Downstream of these recombinant genes, *pR-*hmGST2 and *pR-*hmGST3, we placed an antibiotic-resistance marker gene (Sm^*r*^/Sp^*r*^) for selection. Finally, these cassettes were flanked by *sll1147*-surrounding *Synechocystis* DNA regions to serve as platform of homology for genetic recombination mediating the integration of each DNA cassettes (Δ*sll1147*:*pR-hmGST2-Sm*^*r*^*/Sp*^*r*^ and Δ*sll1147*:*pR-hmGST3-Sm*^*r*^*/Sp*^*r*^) at the *sll1147* locus upon transformation in *Synechocystis*. Similarly, the Δ*sll1147*:*pR-sll0067-Km*^*r*^ control DNA cassette was constructed (Supplementary Table S2 and Supplementary Figure S3) to test the influence of the Sll0067 GST unrelated to Sll1147 [they share a small amino-acids sequence identity percentage (14.4%)] on the presently studied stresses.

The Δ*sll1147*:*pR-hmGST2-Sm*^*r*^*/Sp*^*r*^ and Δ*sll1147*:*pR-hmGST3-Sm*^*r*^*/Sp*^*r*^ cassettes were transformed in the Δ*sll1147*:*Km*^*r*^ selecting for the Sm^*r*^/Sp^*r*^ (Km^*s*^) phenotype. In each case several transformant clones were analyzed by PCR and DNA sequencing (Supplementary Figure S3) to verify that they contained the complementation DNA-cassette Δ*sll1147*:*pR-hmGST2-Sm*^*r*^*/Sp*^*r*^ or Δ*sll1147*:*pR-hmGST3-Sm*^*r*^*/Sp*^*r*^ integrated in place of the Δ*sll1147*:Km^*r*^ locus in all *Synechocystis* chromosome copies, as expected.

**FIGURE 7:**
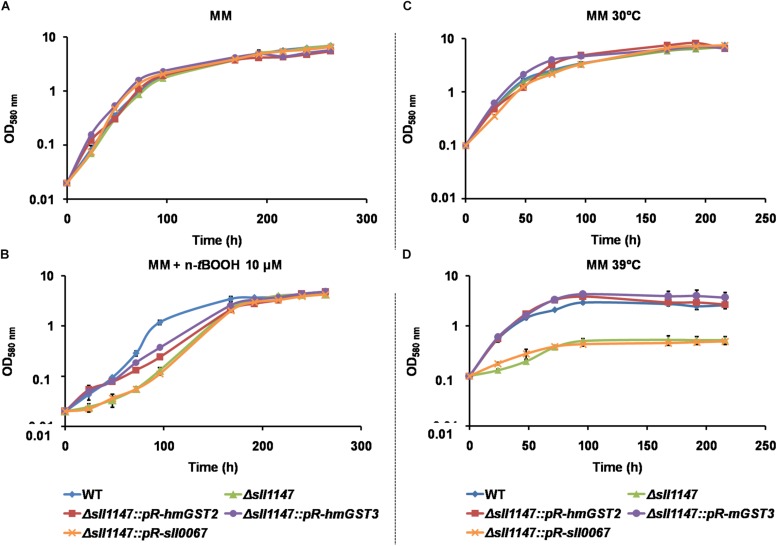
The human hmGST2 and hmGST3 proteins can restore the Δ*sll1147* tolerance to heat and n-*t*BOOH. **(A,B)** Growth curve of *Synechocystis* WT and relevant reporter strains (initial OD_580__nm_ = 0.02) incubated in liquid MM under standard light (2500 lux; 31.25 μE.m^–2^.s^–1^) and temperature (30°C), without **(A)** or with n-*t*BOOH 10 μM **(B)**. **(C,D)** Growth curve of WT and relevant reporter strains (initial OD_580__nm_ = 0.1) incubated in liquid MM under standard light (2500 lux; 31.25 μE.m^–2^.s^–1^) at 30°C **(C)** or 39°C **(D)**. Data shown in all panels are expressed as the mean ± SD of three experiments.

The Δ*sll1147*:*pR-hmGST2-Sm*^*r*^*/Sp*^*r*^ and Δ*sll1147*:*pR-hmGST3-Sm*^*r*^*/Sp*^*r*^ reporter strains grew as healthy as the *Synechocystis* WT strain and the Δ*sll1147*:Km^*r*^ mutant under standard condtions (30°C, Figures 7A,C). Interestingly, the growth of these reporter strains was less affected by the heat (39°C) and n-*t*BOOH (10 μM) stresses than those the Δ*sll1147*:Km^*r*^ mutant (Figures 7A,B). These data indicate that the hmGST2 and hmGST3 human proteins can rescue the tolerance to heat and n-*t*BOOH that are low in the Δ*sll1147*:Km^*r*^ mutant due to its lack of Sll1147 (Figure 7D). To confirm that the higher resistance to n-*t*BOOH and heat of the Δ*sll1147*:*pR-hmGST3-Sm*^*r*^*/Sp*^*r*^ reporter strain was due to the expression of the *pR-hmGST3* gene, not an adventitious selection of a suppressor mutation somewhere in the *Synechocystis* genome, we transformed the Δ*sll1147*:*pR-hmGST3-Sm*^*r*^*/Sp*^*r*^ cells with the Δ*sll1147*:*pR-sll0067-Km*^*r*^ cassette to substitute the *pR-hmGST3* gene by the *pR-sll0067* gene encoding the Sll0067 GST unrelated to the MAPEG proteins hmGST3, hmGST2, and Sll1147/. We verified by PCR and DNA sequencing (Supplementary Figure S3) that the resulting Δ*sll1147*:*pR-sll0067-Km*^*r*^ reporter cells harbored the *pR-sll0067* gene in place of the *pR-hmGST3* gene in every chromosome copy, and that these cells remained as sensitive to n-*t*BOOH and heat as the Δ*sll1147* mutant (Figure 7B), as expected. These data confirmed that the absence of the MAPEG-like Sll1147 protein decreases the tolerance to n-*t*BOOH and heat, which can be rescued by the human orthologs hmGST2 and hmGST3, but not the unrelated cyanobacterial GST Sll0067. Collectively, these finding indicate that MAPEG enzymes, but not all GSTs, play an important role in the cellular resistance to heat and lipid peroxidation, which has been conserved, at least in part, during evolution.

### The Human hmGST2 and hmGST3 MAPEG Proteins Cannot Rescue the Cell Tolerance to Cold, in the *Synechocystis* Mutant Lacking the Sll1147 MAPEG-Like Protein

The same genetic complementation strategy was used to test whether the human hmGST2 and hmGST3 proteins, and the other *Synechocystis* GST protein Sll0067, are able to rescue the cold tolerance of the *Synechocystis* Δ*sll1147*:Km^*r*^ mutant. The survival of the reporter strains Δ*sll1147*:*pR-sll0067* strain was similarly as the Δ*sll1147*:Km^*r*^ deletion mutant suggesting that Sll0067 does not play a role in cold tolerance. The survival of the reporter strains Δ*sll1147*:*pR-hmGST2* and Δ*sll1147*:*pR-hmGST3* were even lower (10-fold) than that of the deletion mutant Δ*sll1147*:Km^*r*^ (Figure 4H) suggesting that both hmGST2 and hmGST3 might play a negative influence on resistance to cold.

### The Human hmGST2 and hmGST3 MAPEG Proteins, but Not the Unrelated Sll0067 *Synechocystis* GST Protein, Can Increase the *E. coli* Tolerance to n-*t*BOOH and Heat

**FIGURE 8:**
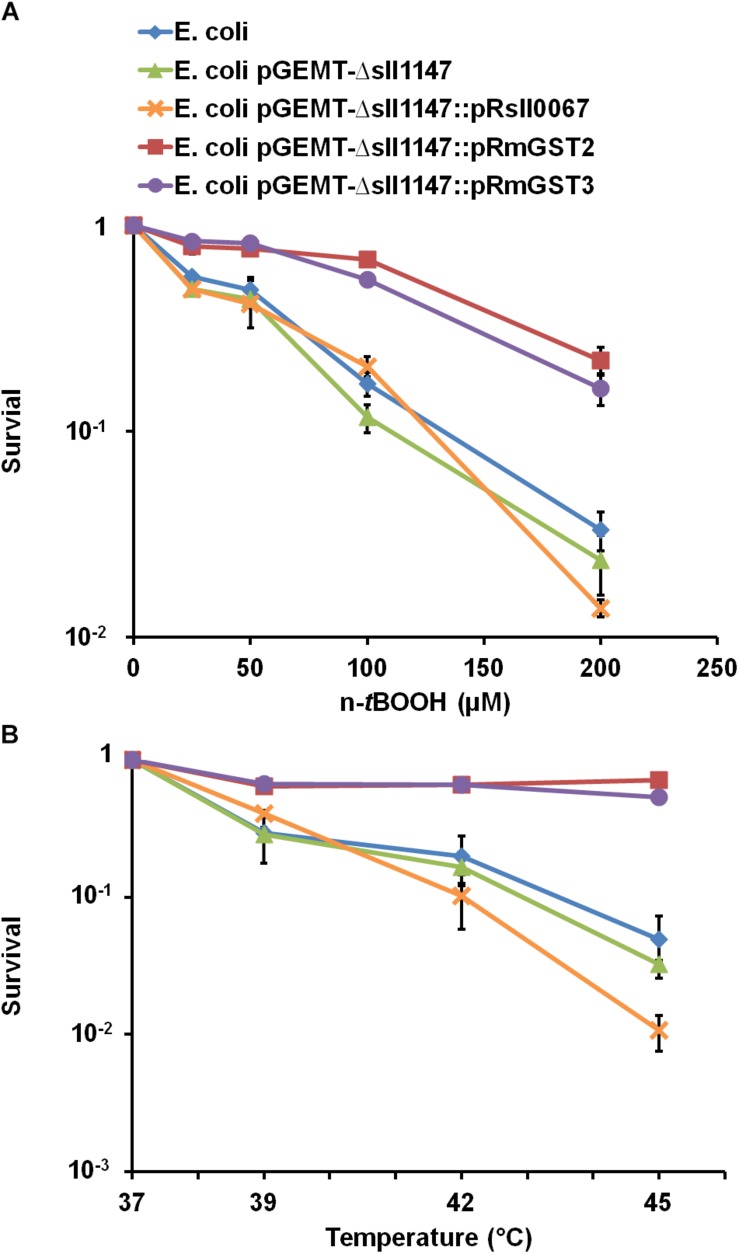
The production of hmGST2 and hmGST3 increase the *Escherichia coli* survival to n-*t*BOOH and heat. Survival of *E. coli* TOP 10 strains harboring, or not (WT control strain) one of the studied GST-producing plasmid challenged for 2 h (initial OD_600__nm_ = 0.1) by the indicated temperatures **(A)** or n-*t*BOOH concentration **(B)**. Data shown in all panels are expressed as the mean ± SD of three experiments.

We also evaluated the influence of the studied protein on the survival of *E. coli* to n-*t*BOOH or high temperatures (Figure 8). As observed in *Synechocystis*, the human MAPEG proteins hmGST2, hmGST3 but not the unrelated cyanobacterial protein Sll0067 could increase the *E. coli* resistance to lipid peroxidation (n-*t*BOOH) and heat. Together the findings in *Synechocystis* and *E. coli* suggest that the MAPEG GSTs play arole in membrane fluidity that has been conserved, at least in part (tolerance to heat and n-*t*BOOH, but not cold) during evolution.

## Discussion

We presently report the first *in vivo* analysis of a MAPEG2-type GST protein, i.e., the protein designated as Sll147 in CyanoBase (see text footnote 1) of the well-studied unicellular cyanobacterium *Synechocystis* PCC 6803 (*Synechocystis*). The motivations for this study are as followins. First, the MAPEG2 sub-family of “Membrane-Associated Proteins involved in Ecosanoïd and Glutathione metabolism” proteins has been poorly studied *in vivo*, even though (i) the human MAPEG2 proteins hmGST2 and hmGST3 are regarded as biomarker for tumorigenesis, and (ii) MAPEG2 proteins are also present in basic easily manipulable organisms like bacteria. Second, only two glutathione transferases (GSTs) have so far been studied *in vivo* in cyanobacteria (Kammerscheit et al., 2019), even though these environmentally crucial prokaryotes are regarded as having developed glutathione (GSH) and GSH-dependent enzymes, such GSTs, to protect themselves against the toxic ROS often produced by their powerful photosynthesis (William Schopf, 2011). These *Synechocystis* Sll1145 and Slr0236 GSTs, unrelated to Sll1147, were shown *in vivo* to play a prominent role in the resistance to photo-oxidative stress (Kammerscheit et al., 2019). Third, Sll1147 and other prokaryotic and eukaryotic MAPEG enzymes produced from recombinant *E. coli* strains were only shown to have the classical *in vitro* GST activity, which is the conjugation of GSH with the standard 1-chloro-2,4-dinitrobenzene (CDNB) exogenous agent (Bresell et al., 2005). Fourth, the analysis of the selectivity/redundancy of GSTs is bound to be easier in a well-studied basic organism such as *Synechocystis* than in higher eukaryotes that possess (i) various tissues (Morgenstern et al., 2011), (ii) complex developmental processes (Björkhem-Bergman et al., 2014) and (iii) large enzyme families. For example, 27 GST encoding genes were identified in human and classified into 7 classes (Alpha, Mu, Pi, Theta, Omega, Zeta, Kappa, and MAPEG) (Nebert and Vasiliou, 2004) whereas *Synechocystis* possesses only six GSTs including Sll1147.

We presently report that the MAPEG-2 type Sll1147 protein is dispensable to the growth of *Synechocystis* in standard photo-autotrophic conditions (Figure 1), whereas it is an important player in cell tolerance heat (Figures 4A–F) and cold (Figures 4G,H), two inevitable environmental stresses for cyanobacteria elicited by the alternation of seasons, the day-night cycles, and the passages of clouds filtering sunlight, which limit their production of biomass that is crucial for our food chain. Using relevant assays, we showed that Sll1147 operates in the protection against peroxidized lipids elicited by heat and cold stresses, using a redox process that transforms reduced glutathione (GSH) in oxidized glutathione (GSSG) (Figures 5, 6). These findings are welcome for the following reason. Though it is known that cold generates a decrease in membrane fluidity, which can be compensated by the desaturation of membrane lipids by fatty acid desaturases, whereas heat causes fluidization of the membranes, which can be compensated by the replacement of unsaturated fatty acids in membrane lipids by newly synthesized saturated fatty acids, these processes are poorly described in cyanobacteria (Maksimov et al., 2017; Pittera et al., 2018). Indeed, to our knowledge, no GST has been proposed to operate in protection against temperature stress in cyanobacteria, yet. Furthermore, cyanobacteria have valuable biotechnological potentials (ecological production of chemicals) that are often hampered by our limited knowledge of their responses to stresses (Jones, 2014; Cassier-Chauvat et al., 2016). Most cyanobacterial species that are being used for chemical production grow optimally between 25 and 35°C, while temperatures in outdoor photobioreactors can be greater than 40°C in subtropical zones. Furthermore, culturing in elevated temperatures can be beneficial in reducing the potential for microbial contamination (Kitchener and Grunden, 2018). Thus, it is important to get a good understanding of heat tolerance in cyanobacteria in the future view order to select or generate strains capable to flourish under high temperatures.

We also showed that Sll1147 is involved in the tolerance to n-*tert*butyl hydroperoxide (n-*t*BOOH), which elicits peroxidized lipids (Johansson et al., 2010), using a redox process that transforms GSH in GSSG (Figures 2, 3), again possibly to restore membrane fluidity. These data are consistent with previous observations that (i) the human hmGST1 protein is active (*in vitro*) on various oxidized lipids (Mosialou et al., 1995); (ii) cancer cells overproducing hmGST1 are more resistant to n-*t*BOOH than their control counterpart (no overproduction of mGST1) (Johansson et al., 2010); and (iii) the sea cucumber mGST1 is also involved in the tolerance to lipid peroxidation (Zhang et al., 2017).

Sll1147 was also found to be required for the tolerance to hydrogen peroxide (H_2_O_2_) (entary Supplementary Figure S1) similarly to what found for the Sll1145 GST (Kammerscheit et al., 2019), unrelated to Sll1147. However, the H_2_O_2_ protection process involving Sll1147 is not accompanied by change in glutathione status (Supplementary Figure S2), unlike what found in the case of Sll1145 (Kammerscheit et al., 2019). Together, these findings emphasize on the selectivity of cyanobacteria GSTs, a poorly investigated field so far.

Finally, as a previous phylogenetic analysis has shown that Sll1147 resembles the human MAPEG hmGST2 and hmGST3 (Bresell et al., 2005), we tested whether the expression of synthetic hmGST2 and hmGST3 genes, adapted to the cyanobacterial codon usage, could restore the tolerance to n-*t*BOOH, and heat in the *Synechocystis* mutant lacking Sll1147 (Δ*sll1147*). Indeed, these complementation tests were positive, whereas the control test performed with the other *Synechocystis* GST Sll0067, unrelated to Sll1147, was negative, as expected (Figure 7). Similar findings were observed when these cyanobacterial and human genes were expressed in *E. coli* (Figure 8). Collectively, these findings indicate that the activity of the MAPEG2 proteins have been conserved, at least in part, during evolution from (cyano)bacteria to human. In this context it is important to note that human MAPEG proteins play a role in inflammation and fever (Sjögren et al., 2013; Bankova et al., 2016, 99) that should be fully investigated. Also interestingly, these human MAPEGs appeared to be able to restore the tolerance of the *Synechocystis*Δ*sll1147* mutant to heat but not cold, which increases or decrease the fluidization of cell membranes, respectively. These findings suggest that depending on their amino-acids sequence MAPEG protein can differently influence some players operating in the signaling of and/or protection against heat but not cold.

## Materials and Methods

### Bacterial Strains, Growth, and Stress Assays

*Escherichia coli* TOP10 (Invitrogen) used for gene manipulation was grown on LB at 37°C. Antibiotic selections were performed with ampicillin (Amp) 100 μg.mL^–1^, kanamycine (Km) 50 μg.mL^–1^ or both streptomycin (Sm) 25 μg.mL^–1^ and spectinomycin (Sp) 75 μg.mL^–1^. For survival analysis, cells challenged by the indicated treatments were plated on Difco LB agar and incubated one day before counting the surviving colonies.

*Synechocystis* PCC 6803 (*Synechocystis*) was grown at 30°C in liquid mineral medium (MM), i.e., BG11 medium (Rippka et al., 1979) enriched with 3.78 mM Na_2_CO_3_ (Jittawuttipoka et al., 2013), under continuous agitation (140 rpm) and white light (2500 lux; 31.25 μE.m^–2^.s^–1^) at 30°C unless stated otherwise. Antibiotic selections were performed with Km 50 μg.mL^–1^ or both Sm 5 μg.mL^–1^ and Sp 5 μg.mL^–1^. For growth analysis mid-exponential phase cultures (OD_580__*nm*_ = 0.3 to 0.8) were adjusted to OD_580__*nm*_ = 0.02 (5.10^5^ cells.mL^–1^) and subsequently incubated in liquid MM containing (or not) the indicated agents, under the indicated temperatures, prior to measuring OD_580_ or photographing the flasks culture. For growth analysis, or OD_580__*nm*_ = 0.1 (2.5 × 10^6^ cells.mL^–1^) for survival analysis, For survival analysis mid-exponential phase cultures were adjusted to OD_580__*nm*_ = 0.1 (2.5 × 10^6^ cells.mL^–1^) before the indicated challenges. Then cells were serially diluted in MM, spread on MM solidified with 1% agar (Difco) and incubated during 5–7 days under standard conditions (2500 lux; 31.25 μE.m^–2^.s^–1^; 30°C) before counting the surviving colonies.

### Construction of the DNA Cassette for Targeted Deletion of the *sll1147* Gene, and Its Substitution by Recombinant Genes Encoding Other GSTs

The two *Synechocystis* DNA regions (about 300 bp in length each) flanking the *sll1147* protein coding sequence (CS) were independently amplified by PCR, using specific oligonucleotides primers (Supplementary Table S1). Then, these two DNA regions were joined by standard PCR-driven overlap extension (Heckman and Pease, 2007) in a single DNA segment harboring a *Sma*I restriction site in place of the *sll1147* CS. After cloning in pGEMt (Promega), the resulting plasmid pGEMT-ABsll1147 (Supplementary Table S2) was opened at the unique *Sma*I site where we cloned the Km^*r*^ cassette (a *Hin*cII fragment of the commercial pUC4K plasmid, Pharmacia) in the same orientation as the *sll1147* CS it replaced. The Δ*sll1147*:Km^*r*^ cassette was transformed (Labarre et al., 1989) to *Synechocystis* wild-type (WT) strain, generating the Δ*sll1147*:Km^*r*^ deletion mutant. The Δ*sll1147*:Km^*r*^ deletion cassette was verified by PCR and nucleotide sequencing (Mix2Seq Kit, Eurofins Genomics) before and after propagation in *Synechocystis*.

We also cloned in the *Sma*I site of pGEMT-ABsll1147 various DNA cassettes that were assembled using NEBuilder HiFi DNA Assembly Master Mix (New England BioLabs) from relevant DNA sequences. These DNA cassette comprises (from 5′ terminus to 3′ end) the very active lambda phage pR promoter and *cro* ribosome binding site (Mermet-Bouvier and Chauvat, 1994) we routinely used for high-level gene expression [see (Ortega-Ramos et al., 2014) and references therein]. These pR promoter and *cro* RBS direct the expression of the synthetic genes (adapted to the *Synechocystis* codon usage for efficient translation) encoding either the human hmGST2 or hmGST3 proteins (Uniprot-ID: Q99735; O14880) or the *Synechocystis* Sll0067 (Uniprot-ID: Q55139) protein. Each recombinant gene was followed by an antibiotic resistant marker gene Km^*r*^ (originating from pUC4K, Pharmacia) or Sm^*r*^/Sp^*r*^ [originating from our pFC1 plasmid (Mermet-Bouvier and Chauvat, 1994)]. All DNA cassettes were verified by PCR and nucleotide sequencing (Mix2Seq Kit, Eurofins Genomics) before and after propagation in *Synechocystis*. The Δ*sll1147*:Km^*r*^ deletion mutant was used as the recipient strain for the transformation by the DNA cassettes required to test the heterologous complementation by the human proteins hmGST2 and hmGST3. The Δ*sll1147*:pR-*sll0067*-Km^*r*^ cassette used to test the possible complementation by Sll0067 was transformed into the Δ*sll1147*:pR-*hmGST3*-Sm^*r*^/Sp^*r*^ mutant.

### Cell Culture and Assay of the Reduced (GSH) and Oxidized (GSSG) Forms of Glutathione

All reagents were purchased from Sigma-Aldrich. Fifty milliliters of exponentially-growing cultures were diluted twofold down to OD_580_ = 0.4, and incubated for various durations under white light (2500 lux; 31.25 μE.m^–2^.s^–1^) in the presence of the indicated agents. Cells were rapidly collected by filtration on a 0.45 μm cellulose membrane (Millipore) under light; re-suspended in 1 mL of acidic extraction phosphate buffer [100 mM KH_2_PO_4_/K_2_HPO_4_; 1 mM EDTA; 5% (w/v) 5-sulfosalicylic acid (SSA)]; disrupted by a three freezing-thawing cycles in liquid nitrogen and hot water bath, and strong mixing (Vibrax VXR, Ika) for 10 min at 4°C; prior to centrifugation (14,000 rpm, 4°C, 5 min) to eliminate unbroken cells and membranes. Cell extracts were purified by a 20 min centrifugation at 14,000 rpm at 4°C through a filter (Amicon Ultra – 0.5 mL 30K; Millipore) to eliminate proteins larger than 30 kDa, and stored at −80°C until use. Before GSSG assay, 100 μL of filtrate were treated with 2 μL of neat 2-vinylpyridine for 1 h on ice to block reduced glutathione (GSH) and then with 2 μL of fourfold diluted triethanolamine solution. For assays, 10 μL untreated filtrate samples (total glutathione assay) and 20 μL treated samples (oxidized GSSG assay) were loaded on a UV-compatible 96-well plate (Greiner bio-one). A first reaction mixture containing yeast GR at final concentration 1.25 U/mL in phosphate buffer (100 mM KH_2_PO_4_/K_2_HPO_4_ buffer and 1 mM EDTA, pH 7.5) was prepared and distributed to each well. A second reaction mixture containing 0.2 mM DTNB [5,5′-dithiobis-(2-nitrobenzoic acid)] and 0.3 mM NADPH in phosphate buffer was automatically added in each well by a microplate reader (ClarioStar; BMG Labtech). The reaction was immediately followed by measuring for 1 min at 30°C the absorption at 412 nm of the yellow TNB (5′-thio-2-nitrobenzoic acid) product (Akerboom and Sies, 1981). Standard curves prepared with various concentrations of GSH or GSSG were used to calculate the GSSG (oxidized) and total glutathione (GSSG + reduced GSH) using the *Synechocystis* cell volume value of 1.2.10^–11^ mL (Mazouni et al., 2004). The GSH content was calculated by subtracting the GSSG content from the total glutathione content.

### Estimation of the Peroxidized Lipids by TBARS Assay

The determination of TBARS as a marker of lipid peroxidation is based on the reaction of MDA and thiobarbituric acid (TBA) in acidic medium at high temperature (Zeb and Ullah, 2016). All chemicals (butylated hydroxytoluene BHT, MDA, and thiobarbituric acid TBA) were purchased from Sigma-Aldrich. 100 mL of exponentially growing cultures were diluted twofold down to OD_580_ = 0.5, and incubated under white light (2500 lux; 31.25 μE.m^–2^.s^–1^) in the indicated conditions. Cells were rapidly collected by filtration as described above and re-suspended in 1 mL of glacial acetic acid supplemented with 0.5 mM BHT (butylated hydroxytoluene BHT) to prevent any further oxidation. MDA extraction was performed as described above for the glutathione assay thanks to: (i) freeze-thaw cycle (liquid nitrogen-hot water), (ii) strong mixing for 10 min at 4°C (Vibrax VXR, Ika) and (iii) centrifugation (14,000 rpm, 4°C, 5 min) to eliminate unbroken cells and membranes. Cells extracts containing MDA were then purified by a 20 min centrifugation at 14,000 rpm at 4°C through a filter (Amicon Ultra – 0.5 mL 30K; Millipore) to eliminate proteins and avoid cross reaction. Filtrates were stored at −80°C until assays in the presence of 100 μL of 4 mM TBA. The absorbance of this mixture was measured at 532 nm using the above-mentioned microplate reader prior to the formation of the MDA-TBA adducts that begins at high temperature (A_532__nm before_). Then, the reaction mixture was heated at 50°C for 3 h and subsequently cooled at room temperature. The absorbance at 532 nm was measured after the reaction (A_532__nm after_) to determine ΔA_532__nm_ = A_532__nm after_ – A_532__nm before_ as the absorbance only due to MDA absorption at 532 nm. In parallel, MDA standard curve was prepared and used to calculate the MDA content using the above-mentioned *Synechocystis* cell volume.

## Data Availability Statement

All datasets generated for this study are included in the manuscript/Supplementary Files.

## Author Contributions

CC-C and FC conceived the project. XK, CC-C, and FC conceived the experiments, analyzed the data, and wrote the manuscript. XK performed the experiments. CC-C agreed to serve as the author responsible for contact and ensures communication.

## Conflict of Interest

The authors declare that the research was conducted in the absence of any commercial or financial relationships that could be construed as a potential conflict of interest.
