# Supplementary material for: From Cyanobacteria to Human, MAPEG-Type Glutathione-S-Transferases Operate in Cell Tolerance to Heat, Cold, and Lipid Peroxidation
Source: Front Microbiol. 2019 Sep 27;10:2248. doi: 10.3389/fmicb.2019.02248 (PMC6798054; doi:10.3389/fmicb.2019.02248)
Supplement: Supplementary file 1 [file Data_Sheet_1.docx]

Supplementary Material

| **Supplementary Table S1. List of the PCR primers used in this study.** | | |
| --- | --- | --- |
| **Amplification of the Km^r^ cassette** | | |
| KmHincII-FW | GGCGCTGAGGTCGACCTCGTGAAGAAG | Amplification of Km^r^ cassette to be cloned as a *Hinc*II fragment |
| KmHincII-RV | ACCTGCAGGGGGTCGACGGAAAGCCAC |  |
| **Amplification of the Km^r^ cassette** | | |
| KmFW | GGTGTTATGAGCCATATTCAACGGG | Amplification of Km^r^ cassette to check the chromosome segregation |
| KmRV | GGGAAGATGCGTGATCTGATCCTTC |  |
| **Amplification of the Sm^r^/Sp^r^ cassette** | | |
| Sm/Sp-FW | ATCTCGAACCGACGTTGCTG | Amplification of Sm^r^/Sp^r^ cassette to check the chromosome segregation |
| Sm/Sp-RV | CCGACTACCTTGGTGATCTC |  |
| **Inactivation of *sll1147*** | | |
| L1147-FWa | CAGGATAGCGGCGGAAAT | Amplification of the *sll1147* downstream region and simultaneous introduction of a *Sma*I site |
| L1147-RVa | ACAGTTCCCGGGACTTTTTGTCATCGGTCATGGT |  |
| L1147-FWb | AGTCCCGGGAACTGTCCCAGTTTTGAACA | Amplification of the *sll1147* upstream region and simultaneous introduction of a *Sma*I site |
| L1147-RVb | GTACAAAGGCATTTTGGATGTG |  |
| SLL1147-FW | GCAACTGCCAAAGGATGGACAGCAG | Amplification of a fragment of *sll1147* gene to check *sll1147* segregation |
| SLL1147-RV | GCTTTGGCGACGATGCTTTACCTGG |  |
| **Construction of complementation cassette** | | |
| pR-A1147-FW | **ACCATGACCGATGACAAAAAGTCCC**ACACCGTGCGTGTTGAC | Amplification of the synthetic over-expressed *hmGST2* and *hmGST3* gene **with DNA platform for DNA assembly** |
| pR-hmGST2-Sm/Sp-RV | **GGTTCTGGACCAGTTGCGTGAGCGC**TTAAAATTGCCGCCGCA |  |
| pR-hmGST3-Sm/Sp-RV | **GGTTCTGGACCAGTTGCGTGAGCGC**TTAATGACAACATTTGG |  |
| Sm/Sp-FW | GCGCTCACGCAACTGGTCCA | Amplification of Sm^r^/Sp^r^ cassette from pFC1 plasmid **with DNA platform for DNA assembly** |
| Sm/Sp-B1147-RV | **TTTGTTCAAAACTGGGACAGTTCCC**CTCCCAATTTGTGTAGGGCT |  |
| SLL0067-FW | ATGATCAAACTATACGGTGC | Amplification of the *sll0067* gene |
| SLL0067-RV | TCAGCGGGCACCGATGGAAGC |  |
| A1147-pR-pGEMt-FW | **TCCCGGCCGCCATGGCCGCGGGATT**CAGGATAGCGGCGGAAATATAGGCC | Amplification of the upstream region of the *sll1147* gene **with DNA platform for DNA assembly** |
| A1147-pR-sll0067-RV | **GGGGGGCACCGTATAGTTTGATCAT**ATGACCTCCTTAGTACATGCAACC |  |
| Km-sll0067-FW | **GCTTCCATCGGTGCCCGCTGA**GGAAAGCCACGTTGTGTCTC | Amplification of Km^r^ cassette **with DNA platform for DNA assembly** |
| Km-B1147-FW | **CACAAAGTTTGTTCAAAACTGGGACAGTT**CCCGGCGCTGAGGTCTGCCTCG |  |
| B1147-FW | GGGAACTGTCCCAGTTTTG | Amplification of the downstream region of the *sll1147* gene **with DNA platform for DNA assembly** |
| B1147-pGEMt-RV | **CAGGCGGCCGCACTAGTGATT**GTACAAAGGCATTTTGGATGTG |  |
| **Abbreviations:** FW: forward; RV: reverse | | |

**Supplementary Table S2. Characteristics of the plasmids constructed in this study.**

| **Plasmid** | **Relevant features** | **Reference** |
| --- | --- | --- |
| **pGEMT** | Amp^r^ AT overhang cloning vector | Promega |
| **pUC4K** | Source of the Km^r^ marker gene | Pharmacia |
| **pFC1** | Source of the Sm^r^/Sp^r^ marker gene | * |
| **pGEMT-AB*sll1147*** | pGEMT with the downstream and upstream flanking sequences of *sll1147* gene, where the most of the *sll1147* coding sequences (from the 24^th^ bp downstream of the ATG codon down to the 26^th^ bp upstream of the stop codon up) was replaced by a *Sma*I restriction site | This study |
| **pGEMT-Δ*sll1147*::Km^r^** | pGEMT-AB*sll1147* with the Km^r^ marker inserted in the unique *Sma*I site | This study |
| **pEX-pR*hmGST2*** | pEX-A128 plasmid (Eurofins genomics) which contains the **synthetic** gene pR-*hmGST2:*  acaccgtgcgtgttgactattttacctctggcggtgataatggttgc**a**tgtactaaggaggtCAT**ATGGCCGGCAATTCCATTTTGTTGGCCGCCGTGTCCATTTTGTCCGCCTGTCAACAATCCTATTTTGCCTTGCAAGTGGGCAAAGCCCGGTTGAAATATAAAGTGACCCCCCCCGCCGTGACCGGCTCCCCCGAATTTGAACGGGTGTTTCGGGCCCAACAAAATTGTGTGGAATTTTATCCCATTTTTATTATTACCTTGTGGATGGCCGGCTGGTATTTTAATCAAGTGTTTGCCACCTGTTTGGGCTTGGTGTATATTTATGGCCGGCATTTGTATTTTTGGGGCTATTCCGAAGCCGCCAAAAAACGGATTACCGGCTTTCGGTTGTCCTTGGGCATTTTGGCCTTGTTGACCTTGTTGGGCGCCTTGGGCATTGCCAATTCCTTTTTGGATGAATATTTGGATTTGAATATTGCCAAAAAATTGCGGCGGCAATTTTAA**  Corresponding protein sequence (Uniprot ID = Q99735):  >MGST2_HUMAN Microsomal glutathione S-transferase 2 OS=Homo sapiens  MAGNSILLAAVSILSACQQSYFALQVGKARLKYKVTPPAVTGSPEFERVFRAQQNCVEFY  PIFIITLWMAGWYFNQVFATCLGLVYIYGRHLYFWGYSEAAKKRITGFRLSLGILALLTL  LGALGIANSFLDEYLDLNIAKKLRRQF | This study |
| **pEX-pR*hmGST3*** | pEX-A128 plasmid (Eurofins genomics) which contains the **synthetic** gene pR-*hmGST3:*  acaccgtgcgtgttgactattttacctctggcggtgataatggttgc**a**tgtactaaggaggtCAT**ATGGCCGTGTTGTCCAAAGAATATGGCTTTGTGTTGTTGACCGGCGCCGCCTCCTTTATTATGGTGGCCCATTTGGCCATTAATGTGTCCAAAGCCCGGAAAAAATATAAAGTGGAATATCCCATTATGTATTCCACCGATCCCGAAAATGGCCATATTTTTAATTGTATTCAACGGGCCCATCAAAATACCTTGGAAGTGTATCCCCCCTTTTTGTTTTTTTTGGCCGTGGGCGGCGTGTATCATCCCCGGATTGCCTCCGGCTTGGGCTTGGCCTGGATTGTGGGCCGGGTGTTGTATGCCTATGGCTATTATACCGGCGAACCCTCCAAACGGTCCCGGGGCGCCTTGGGCTCCATTGCCTTGTTGGGCTTGGTGGGCACCACCGTGTGTTCCGCCTTTCAACATTTGGGCTGGGTGAAATCCGGCTTGGGCTCCGGCCCCAAATGTTGTCATTAA**  Corresponding protein sequence (Uniprot ID = O14880):  >MGST3_HUMAN Microsomal glutathione S-transferase 3 OS=Homo sapiens  MAVLSKEYGFVLLTGAASFIMVAHLAINVSKARKKYKVEYPIMYSTDPENGHIFNCIQRA  HQNTLEVYPPFLFFLAVGGVYHPRIASGLGLAWIVGRVLYAYGYYTGEPSKRSRGALGSI  ALLGLVGTTVCSAFQHLGWVKSGLGSGPKCCH | This study |
| pR promotor showing the -35 box (ttgact), the -10 box (gataat) and the transcription start site (A) and the Shine d'Algarno (ribosome bind site; aaggagg) sequence originating from the *cro* gene of lambda phage is fused at the *Nde*I restriction site (CAT**ATG**) with the coding sequence of mGST2 or mGST3 adapted to *Synechocystis* codon usage. | | |
| **pGEMT-Δ*sll1147*::pR*-sll0067*-Km^r^** | pGEMT-AB*sll1147* with the synthetic gene pR*sll0067* and the Km^r^ marker | This study |
| **pGEMT-Δ*sll1147*::*pR-mGST2*-Sm^r^/Sp^r^** | pGEMT-AB*sll1147* with the synthetic gene pR*mGST2* and the Sm^r^/Sp^r^ marker | This study |
| **pGEMT-Δ*sll1147*::pR*-mGST3*-Sm^r^/Sp^r^** | pGEMT-AB*sll1147* with the synthetic gene pR*mGST3* and the Sm^r^/Sp^r^ marker | This study |
| *(Mermet-Bouvier and Chauvat, 1994) | | |


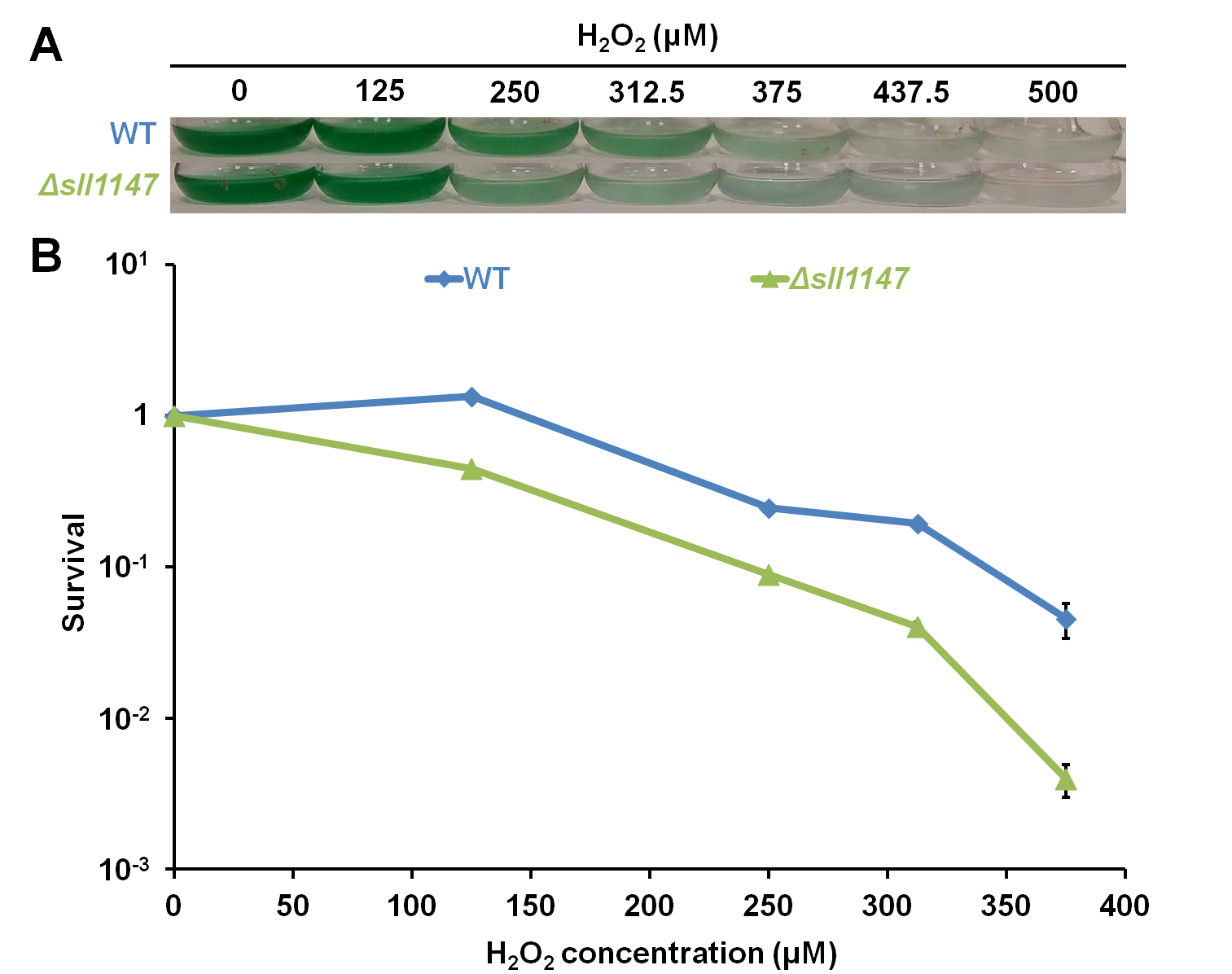


Supplementary Figure S1. The ∆*sll1147* mutant is slightly sensitive to hydrogen peroxide.

(A) Ten milliliters of mid-log-phase cultures (initial OD_580 nm_ = 0.1) were incubated in liquid MM with or without the indicated concentrations of H_2_O_2_ for 72h under 2500 lux (31.25 μE.m^-2^.s^-1^) at 30°C prior to photography culture flasks. (B) Typical survival of the WT and mutant exposed for 72h to various H_2_O_2_ concentrations. Data shown in (B) are expressed as the mean +/- standard deviation (SD) (n=3).


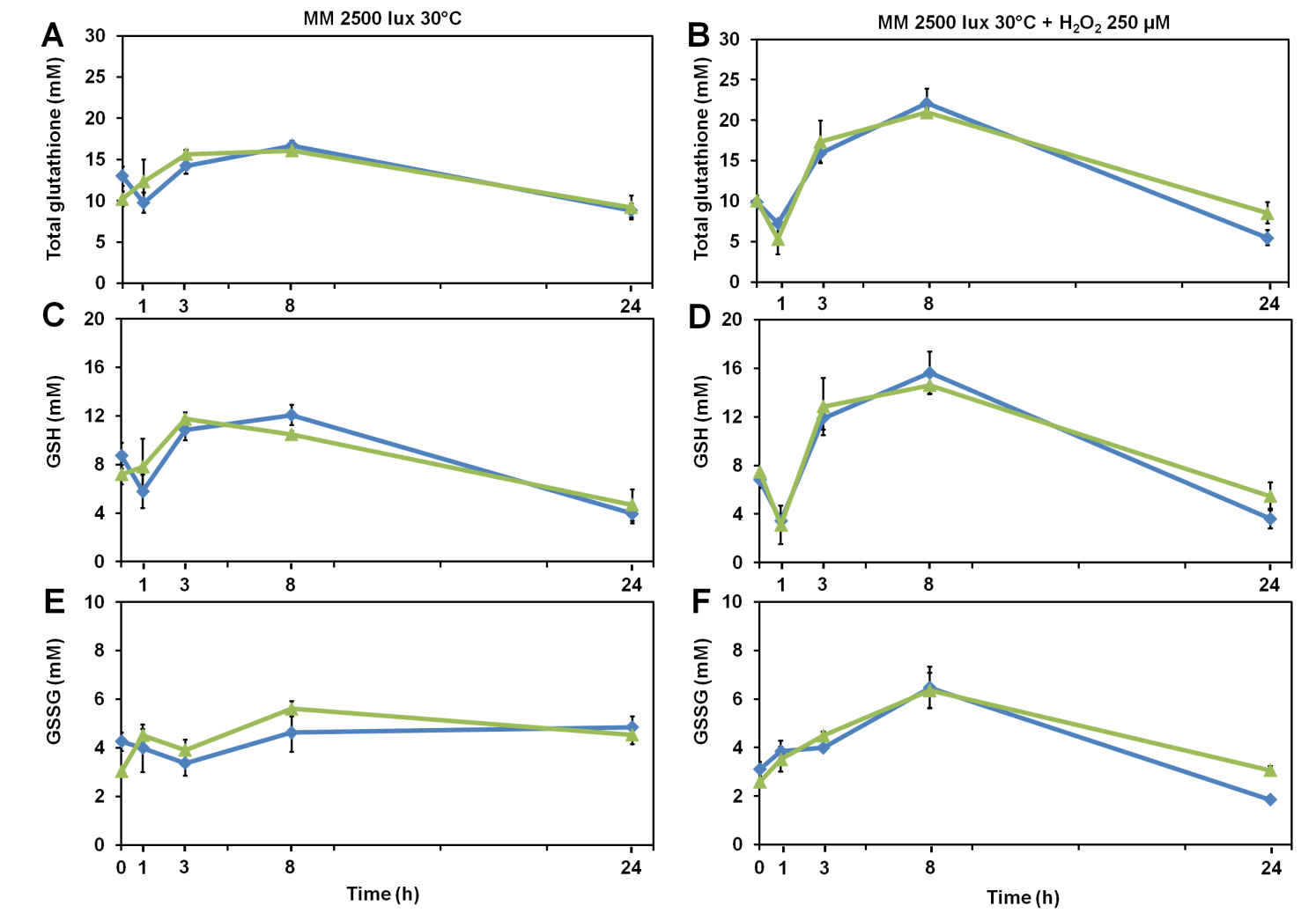


Supplementary Figure S2. In response to H_2_O_2_, ∆*sll1147* and wild-type cells displayed very similar changes of glutathione abundance.

Kinetic analysis of the influence of H_2_O_2_ on the abundance of total glutathione (A-B), GSH (C-D) and GSSG (E-F) in *Synechocystis* WT and mutant ∆*sll1147*. Data are expressed as the mean +/- SD (n=3) and * indicates significant difference between mutant and WT (*t*-test, *P*< 0.05).


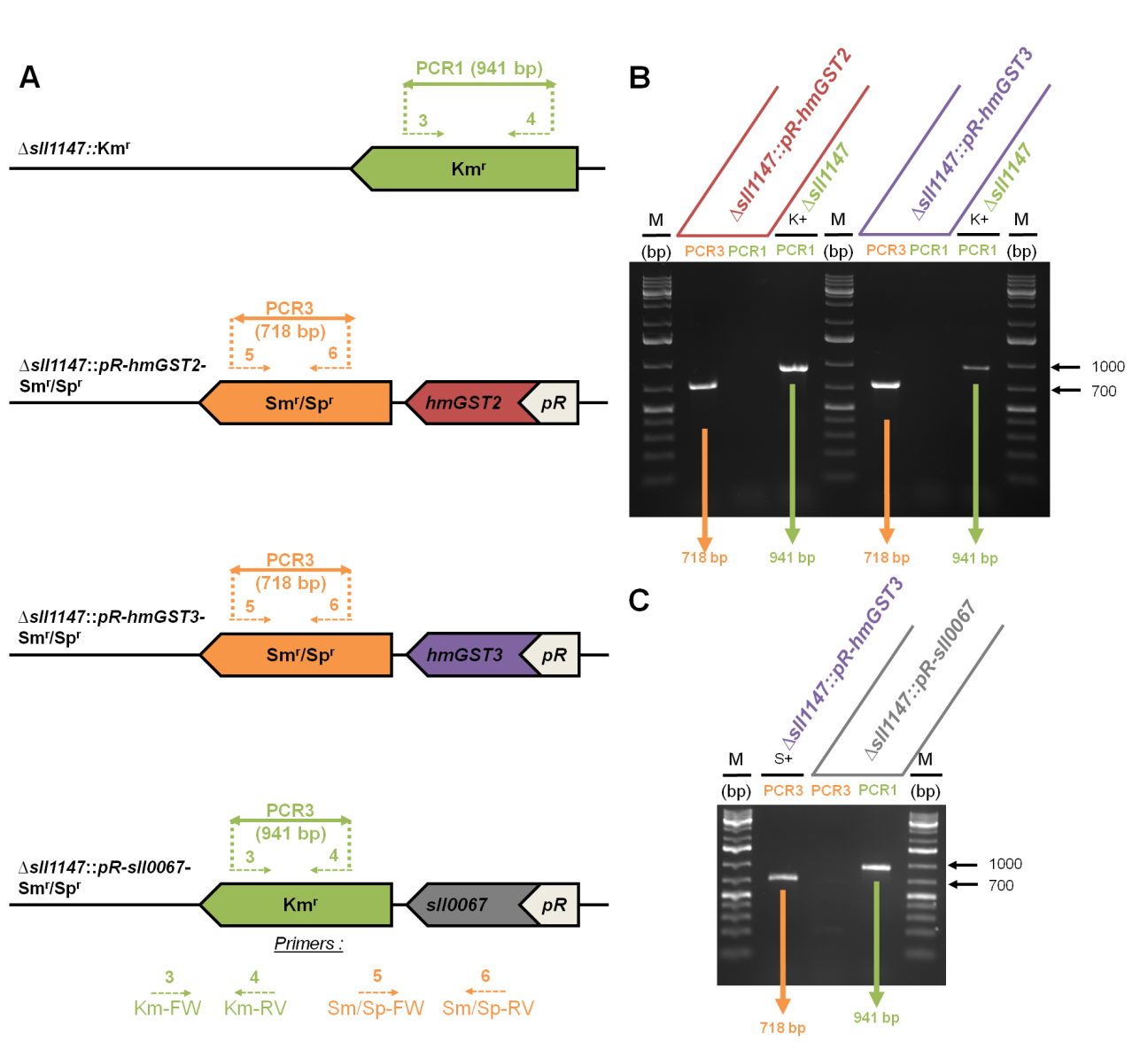


Supplementary Figure S3. Verification that the *sll1147*-complementation DNA cassettes are properly integrated in the Δ*sll1147::Km^r^* chromosome locus.

(A) Schematic representation of the *sll1147* chromosome loci in the Δ*sll1147*::Km^r^ mutant before or after transformation with the gene complementation DNA cassettes Δ*sll1147::pR-hmGST2*-Sm^r^/Sp^r^ or Δ*sll1147::pR-hmGST3*-Sm^r^/Sp^r^ or Δ*sll1147::pR-sll0067*-Km^r^ required to test the ability of the recombinant genes *pR-hmGST2*-Sm^r^/Sp^r^, *pR-hmGST3*-Sm^r^/Sp^r^ and *pR-sll0067*-Km^r^ expressed from the strong *pR* promoter [39] to restore the stress tolerance of the Δ*sll1147*::Km^r^ mutant back to the WT level. The *pR-hmGST2*-Sm^r^/Sp^r^, *pR-hmGST3*-Sm^r^/Sp^r^ cassettes were introduced directly in the Δ*sll1147*::Km^r^ mutant, whereas the *pR-sll0067*-Km^r^ cassette was introduced in the *pR-hmGST3*-Sm^r^/Sp^r^ mutant. The genes are represented by colored arrows (Km^r^: green; Sm^r^/Sp^r^: orange; *hmGST2*: red; *hmGST3*: purple; *sll0067*: grey) that point into the direction of their transcription. The same color code is used to represent the PCR primers (dotted arrows, See S1 Table for their sequence) and the corresponding PCR products (double arrows) typical of the chromosome of the mutants generated in this study Δ*sll1147*::Km^r^ or Δ*sll1147::pR-hmGST2*-Sm^r^/Sp^r^ or Δ*sll1147::pR-hmGST3*-Sm^r^/Sp^r^ or Δ*sll1147::pR-sll0067*-Km^r^. (B and C) Typical UV-light image of the agarose gel showing the PCR products corresponding to the antibiotic resistant markers Km^r^ (PCR1: green), Sm^r^/Sp^r^ (PCR3: orange) generated, or not (negative controls), from the mutants Δ*sll1147*::Km^r^, Δ*sll1147::pR-hmGST2*-Sm^r^/Sp^r^, Δ*sll1147::pR-hmGST3*-Sm^r^/Sp^r^ and Δ*sll1147::pR-sll0067*-Sm^r^/Sp^r^ cells. M (marker DNA) indicates the GeneRuler 1 kb Plus DNA Ladder (Thermo Scientific). Note that all mutants are fully segregated and harbor only one type of chromosome copies.
